# Network-based approaches elucidate differences within APOBEC and clock-like signatures in breast cancer

**DOI:** 10.1186/s13073-020-00745-2

**Published:** 2020-05-29

**Authors:** Yoo-Ah Kim, Damian Wojtowicz, Rebecca Sarto Basso, Itay Sason, Welles Robinson, Dorit S. Hochbaum, Mark D. M. Leiserson, Roded Sharan, Fabio Vadin, Teresa M. Przytycka

**Affiliations:** 1 National Center for Biotechnology Information, National Library of Medicine, National Institutes of Health, 8600 Rockville Pike, Bethesda, 20894 USA; 2 Department of Industrial Engineering and Operations Research, University of California, Berkeley, 94720 CA USA; 3 School of Computer Science, Tel Aviv University, Tel Aviv, 69978 Israel; 4 Center for Bioinformatics and Computational Biology, University of Maryland, 8314 Paint Branch Dr, College Park, 20742 USA; 5 Department of Information Engineering, University of Padova, Via Gradenigo 6/A, Padua, I-35131 Italy

**Keywords:** Mutational signature, Continuous cancer phenotype, Gene network, Network-phenotype association, Breast cancer, APOBEC, Clock-like signatures

## Abstract

**Background:**

Studies of cancer mutations have typically focused on identifying cancer driving mutations that confer growth advantage to cancer cells. However, cancer genomes accumulate a large number of passenger somatic mutations resulting from various endogenous and exogenous causes, including normal DNA damage and repair processes or cancer-related aberrations of DNA maintenance machinery as well as mutations triggered by carcinogenic exposures. Different mutagenic processes often produce characteristic mutational patterns called mutational signatures. Identifying mutagenic processes underlying mutational signatures shaping a cancer genome is an important step towards understanding tumorigenesis.

**Methods:**

To investigate the genetic aberrations associated with mutational signatures, we took a network-based approach considering mutational signatures as cancer phenotypes. Specifically, our analysis aims to answer the following two complementary questions: (i) what are functional pathways whose gene *expression* activities correlate with the strengths of mutational signatures, and (ii) are there *pathways whose genetic alterations* might have led to specific mutational signatures? To identify mutated pathways, we adopted a recently developed optimization method based on integer linear programming.

**Results:**

Analyzing a breast cancer dataset, we identified pathways associated with mutational signatures on both expression and mutation levels. Our analysis captured important differences in the etiology of the APOBEC-related signatures and the two clock-like signatures. In particular, it revealed that clustered and dispersed APOBEC mutations may be caused by different mutagenic processes. In addition, our analysis elucidated differences between two age-related signatures—one of the signatures is correlated with the expression of cell cycle genes while the other has no such correlation but shows patterns consistent with the exposure to environmental/external processes.

**Conclusions:**

This work investigated, for the first time, a network-level association of mutational signatures and dysregulated pathways. The identified pathways and subnetworks provide novel insights into mutagenic processes that the cancer genomes might have undergone and important clues for developing personalized drug therapies.

## Background

Cancer genomes accumulate a high number of mutations, only a small portion of which are cancer driving mutations. Most of such mutations are passenger somatic mutations, not directly contributing to cancer development. Analyses of large-scale cancer genome data revealed that these passenger mutations often exhibit characteristic mutational patterns called “mutational signatures” [1]. Importantly, these characteristic mutational signatures are often linked to specific mutagenic processes, making it possible to infer which mutagenic processes have been active in the given patient. This information often provides important clues about the nature of the diseases. For example, the presence of specific signatures associated with homologous recombination repair deficiency (HRD) can help identify patients who can benefit from PARP inhibitor treatment [2]. With the increased interest in the information on mutagenic processes acting on cancer genomes, several computational approaches have been developed to define mutational signatures in cancer [1, 3–7], to identify patients whose genome contains given signatures [6–8], to map patient mutations to these signatures [9], and to identify superposition of several mutagenic processes [10].

Despite the importance of understanding cancer mutational signatures, the etiology of many signatures is still not fully understood. It is believed that mutational signatures may arise not only as a result from exogenous carcinogenic exposures (e.g., smoking, UV exposures) but also due to endogenous causes (e.g., HRD signature mentioned above). That is, human genomes are protected by multiple DNA maintenance and repair mechanisms in the presence of various types of DNA damage, but aberrations or other malfunctions in such mechanisms can leave errors not repaired, generating specific patterns of mutations [11].

From the perspective of individual patients, it is important to determine mutational signatures imprinted on each patient’s genome and the strength of the (sometimes unknown) mutagenic processes underlining the signatures. Signature strength can be measured by the number of mutations that are attributed to the given signature and thus can be considered as a continuous phenotype. With this view in mind, we investigate the relation of this phenotype with other biological properties of cancer patients. In this study, we focus on the relation of mutational signature strength with gene expression in biological processes and gene alteration in subnetworks.

The hypothesis that mutational signatures can be related to aberrant gene expression or alterations in DNA repair genes is well supported. For example, the deactivation of MUTYH gene in cancer patients is associated with a specific mutational signature [11–13]. Previous studies identified correlations between several mutational signatures and some cancer drivers and acknowledged that the cause-effect relation between signatures and cancer drivers can be in either direction [14]. On the other hand, like many other cancer phenotypes, the causes of mutational signatures can be heterogeneous and the same signature can arise due to different causes. For example, the abovementioned signature caused by the inactivation of the MUTYH gene was also found in cancers that do not harbor this aberration [15]. With the observation that different mutations in functionally related genes can lead to the same cancer phenotype [16–18], cancer phenotypes are increasingly considered in the context of genetically dysregulated pathways rather than in the context of individual genes [19–24]. Hence, we postulated that identifying mutated subnetworks and differentially expressed gene groups that are associated with mutational signatures can provide new insights on the etiology of mutational signatures.

In this study, we focused on mutational signatures in breast cancer, for which a large data set is available, including whole genome mutation profiles as well as expression data [25]. The mutagenic landscape of this cancer type is complex and is yet to be fully understood. For example, previously defined *COSMIC* signatures present in breast cancer [25] include two signatures (Signatures 1 and 5) as age related (clock-like) and two signatures associated with the activities of APOBEC enzyme (Signatures 2 and 13). The mechanisms underlying the differences between two distinct signatures with similar etiology are not fully understood.

The clock-like signatures (COSMIC Signatures 1 and 5) have been found correlated with the age of patients, but the strengths of correlation differ between the two signatures and vary across different cancer types [26]. Signature 1 is considered to arise from an endogenous mutational process initiated by spontaneous deamination of 5-methylcytosine while the etiology of Signature 5 is less understood. Therefore, it is important to understand what processes, other than patient’s age, contribute to each of these signatures.

APOBEC signatures have been the subject of particular attention [27–35]. The proteins encoded by APOBEC gene family (known to be involved in immune response) deaminate cytosines in single-stranded DNA (ssDNA). Such deamination, if not properly repaired, can lead to C >T (Signature 2) or C >G (signature 13) mutations depending on how the resulting lesion is repaired or bypassed during the replication [36]. Thus, the final imprint of APOBEC-related mutations on the genome depends on several factors: expression level of APOBEC genes, the amount of accessible ssDNA, and the lesion bypass mechanism. In particular, clustered APOBEC-induced mutations (*kataegis*) in breast cancer are assumed to be a result of the mutation opportunity offered by single-stranded DNA during repair of double-stranded breaks (DSBs). However, ssDNA regions can also emerge for other reasons such as topological stress. Thus, although several aspects contributing to the APOBEC signatures have been known for some time, we are yet to uncover the full complexity of the APOBEC-derived signatures.

To address these challenges, we took two complementary pathway-based approaches: one focused on gene modules whose expression correlates with signature strength and the second based on the identification of subnetworks of genes whose alterations are associated with mutational signatures.

Our study provides several new insights on the mutagenic processes in breast cancer including (i) association of the NER pathway and oxidation processes with the strength of clock-like Signature 5, (ii) differences between the two clock-like signatures with respect to their associations with cell cycle, and (iii) differences in mutated subnetworks associated with different signatures including APOBEC-related signatures. We demonstrate that our findings are consistent with the results from recent studies and provide additional insights that are important for understanding mutagenic processes in cancer and developing anti-cancer drugs.

## Methods

### Overview

In this study, we consider mutational signatures in cancer patients and attempt to identify genes and pathways whose expression and/or genetic alterations are potentially causative of differences in mutational signature strength. We utilized the somatic mutations in the cohort of 560 breast cancer (BRCA) whole genomes [25]. We used 12 COSMIC signatures indited as active in BRCA in previous studies (Signatures 1, 2, 3, 5, 6, 8, 13, 17, 18, 20, 26, and 30). Since recent studies revealed that mutations occurring in close proximity to each other, referred to here as cloud mutations, have distinct properties from dispersed mutations [9, 37], we additionally subdivided all mutations (and subsequently their attributed signatures) into two groups—close-by Cloud mutations and Dispersed mutations (see the “Data” section)

**Fig. 1:**
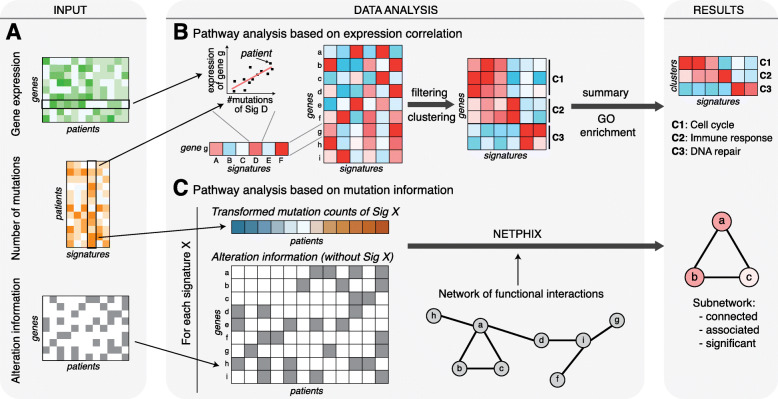
Overview of the study. **a** The input data for this study consist of gene expression, mutational signature counts, and gene alteration across a number of cancer patients. **b** The functional pathways whose gene expression levels are associated with mutational signatures were found by computing correlations between expression levels of all genes and signature mutation counts, filtering out weak correlations, clustering expression correlation profiles, and performing GO enrichment analysis of the identified clusters. **c** The pathways whose gene alterations are associated with mutational signatures were found by applying NETPHIX to the transformed signature mutation counts (*z*-score of log-transformed counts), gene-patient alteration matrix, and a known functional interaction network

In the first part of the analysis, we looked for the genes whose expression levels are significantly correlated with mutational signature strength (Fig. 1a, b). Specifically, we first selected genes exhibiting significant correlation with at least one mutational signature by computing the correlation coefficient of the expression profile and mutation counts for each pair of genes and signatures. The selected genes were clustered based on their expression correlation patterns across mutational signatures (see the “Expression correlation analysis” section).

The second part of the analysis involves uncovering subnetworks of genes whose alterations are associated with mutational signature strength (Fig. 1a, c). We hypothesize that a certain mutational signature can arise when a related pathway (e.g., DNA damage repair mechanism) is dysregulated. Due to the complex nature of cancer driving mutations, we adapted the NETPHIX method—a recently developed network-based method to identify mutated subnetworks associated with continuous phenotypes [38]—to identify such pathways. In this analysis, we consider the mutation count of a mutational signature in a whole cancer genome to be a cancer phenotype and aim to identify a subnetwork of genes whose alterations are associated with the phenotype. Importantly, when assessing association between gene-level alterations and a mutational signature, the mutations attributed to the given mutational signature were not incorporated into the alteration information (Fig. 1c; the “Mutation analysis” section, and Additional file 1: Supplemental Methods) in order to increase the likelihood of uncovered subnetworks being drivers of the signatures rather than their effect.

### Data

We analyzed the somatic mutations in the cohort of 560 breast cancer (BRCA) whole genomes published by Nik-Zainal et al. [25]. The mutation data (single base substitutions and small indels) were downloaded from the ICGC data portal (release 22) [39]. The most likely assignments of 3,479,652 individual point mutations to mutational signatures were generated with SIGMa [9] using 12 predefined COSMIC signatures (version 2; https://cancer.sanger.ac.uk/cosmic/signatures_v2) known to be active in BRCA (Signatures 1, 2, 3, 5, 6, 8, 13, 17, 18, 20, 26, and 30) [25]. SIGMa is a probabilistic model of sequential dependency for mutation signatures that allows for an accurate assignment of mutations to predefined signatures (it does not infer new signatures). To ensure SIGMa’s robustness with respect to random initialization used in its learning process, we computed the majority assignments over 31 random initialization runs. SIGMa relies on the observation that adjacent mutations in a given cancer genome are more likely to be the result of the same mutation signature and that mutations that are assigned to the same signature can have distinct properties when being isolated versus being localized in clusters [25, 36, 37]. Thus, it divides all mutations into two groups—close-by (clustered) **C**loud mutations and **D**ispersed (sky) mutations. The sequential dependencies between close-by mutations are modeled by a Hidden Markov model, while for dispersed mutations, we use a multinomial mixture model. Here, we treat cloud and dispersed mutations, and their associated signatures, separately. For each patient, we computed signature profiles based on the patient mutation counts assigned to each specific signature, separating cloud and dispersed mutations. The mutational signature profiles were used as phenotype profiles in the expression correlation and mutated pathway analyses (Fig. 1a). For further analysis, we used only sufficiently abundant mutational signatures for cloud or dispersed mutations whose overall exposure levels are above 10% within both groups of mutations. This created 10 different phenotype profiles for Signatures 1D, 2C/D, 3C/D, 5D, 8C/D, and 13C/D, where the numbering refers to the COSMIC signature index and C/D denotes signatures attributed to close-by cloud and dispersed mutations.

### Expression correlation analysis

**Fig. 2:**
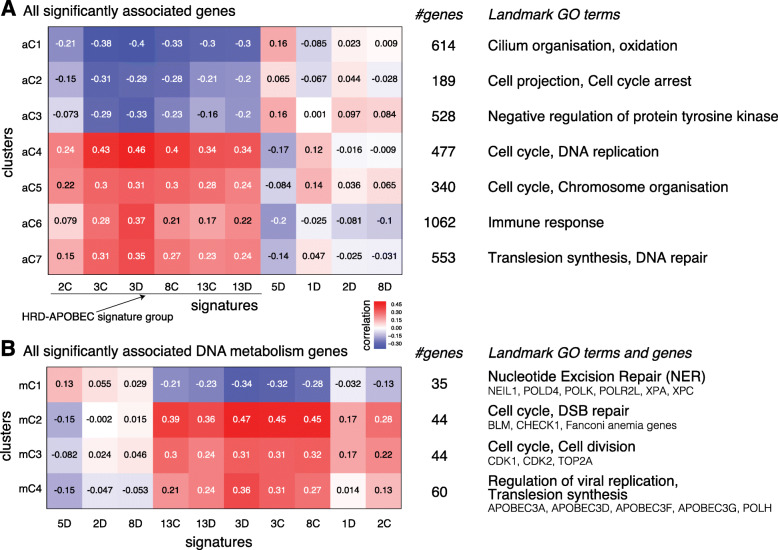
Gene expression correlation modules. **a** All genes significantly correlated with at least one signature (|*corr*|≥0.3 and adjusted *pv*≤0.005). **b** DNA metabolic process genes, based on Gene Ontology (GO), significantly correlated with at least one signature. For both (**a** and **b**), we show a heatmap of mean expression correlation for each cluster and signature (left), number of genes in each cluster (middle), and representative GO terms enriched in cluster genes (right). For the DNA metabolic process, we also show representative genes for each cluster. The list of genes and GO enrichment terms for the clusters is provided in Additional file 2: Table S2 and Additional file 3: Table S3

To identify expression-based pathways that are associated with signatures, we downloaded the normalized gene expression data for 266 BRCA patients from Supplementary Table 7 of Nik-Zainal et al. [25] and used correlation analysis followed by clustering of correlation patterns. Specifically, we first computed the Spearman correlation coefficient of the expression level and mutation count for each pair of genes and mutational signatures. We then selected the genes exhibiting significant correlation with at least one of 10 mutational signatures; the expression of a gene is considered significantly correlated with a signature if |*corr*|≥0.3 and adjusted *pv*≤0.005 (*corr* is Spearman correlation coefficient, BH-corrected *p*value). The procedure selected 3763 genes. We then clustered the genes based on their correlation pattern using a consensus K-means algorithm: running K-means clustering 100 times with random start and varying *k* from 5 to 50 and subsequently running hierarchical clustering with consensus matrix from 100 runs of K-means. GO enrichment analysis was performed using hypergeometric test, and significant terms were selected with nominal *p*value < 0.05. The final 7 clusters and enrichment analysis results are summarized in Fig. 2a and Additional file 2: Table S2 (more fine-grained results with 12 clusters are also shown in Additional file 1: Fig. S1). The source code and data files are available at Github [40].

To take a closer look at DNA repair genes, we performed similar analysis with genes in GO DNA metabolic process. One hundred eighty-four genes are selected with the same significance cutoffs. The hierarchical clustering of the consensus clustering for 100 K-means (*k*=2 to 20) generated 4 clusters shown in Fig. 2b and Additional file 3: Table S3. The enrichment analysis was performed using hypergeometric test with only the genes in GO DNA metabolic process as the background, and only for the GO terms with significant overlaps with GO DNA metabolic process (at least 2 genes in common and *p*value of the intersection <0.05).

### Mutation analysis

To find alteration-based pathways for signatures, we adapted a recently developed method, NETPHIX, which identifies mutated subnetworks associated with a continuous phenotype [38]. Given gene alteration information of cancer samples and continuous phenotype values for the same samples, NETPHIX aims to identify a connected subnetwork whose aggregated alterations are associated with the phenotype of interest (mutation counts for cancer mutational signatures in this study). NETPHIX utilizes functional interaction information among genes and enforces the identified genes to be *connected* in the network while, at the same time, making sure that the aggregated alterations of these genes are significantly *associated* with the given phenotype. In addition, in its integer linear program formulation, NETPHIX recognizes that cancer driving mutations tend to be mutually exclusive [22, 41–45] and incorporates this property in its objective function [38]. The detailed description of NETPHIX is given in Additional file 1: Supplemental Methods.The source code and data files for NETPHIX analysis are available at Github [40].

For the gene-level alteration information (the bottom matrix in Fig. 1a), we utilized all somatic point mutations and small indels for the same 560 patient data. In processing the somatic mutation data, we defined a gene to be altered if it has at least one non-silent mutation in its genomic region. In addition to somatic mutations, DNA repair genes can undergo alternative mechanisms of inactivation including pathogenic germline variants and promoter hypermethylation. A recent paper highlighted the importance of these mechanisms in inactivating the homologous recombination pathway [2]. To account for these additional sources of inactivation, we also defined a gene to be altered in a patient if the gene is annotated as being biallelic inactivated for the patient in Supplementary Tables 4a and 4b of Davies et al. [2]. The gene alteration information is used to find mutated subnetworks associated with each signature (Fig. 1c). When computing association with a specific signature, we further refined the information to increase the likelihood that the association is causative (i.e., gene alteration causes mutational signatures, not vice versa). Specifically, the gene alteration information for the association analysis with a specific mutational signature was constructed after excluding the mutations attributed to the given mutational signature (see Additional file 1: Supplemental Methods for details). Similarly, we removed all indels when we considered the associations with Signatures 3 and 8 as these signatures are believed to lead to a high burden of indels. The assignment of mutations to signatures was performed using SIGMa (see above).

For each mutational signature, we normalized the mutation counts by taking log and subsequently computing *z*-scores and used the profiles as phenotype inputs to NETPHIX. For functional interactions among genes, we used the data downloaded from STRING database version 10.0 [46], only including the edges with high confidence scores (≥900 out of 1000). The alteration tables were constructed as described above, and genes altered in less than 1% of patients were removed from further consideration. We ran NETPHIX for each mutational signature with density constraint of 0.5 and for a fixed size modules *k* from 1 to 7. The appropriate *k* was selected by examining the increase of the objective function values and the significance of the solution using permutation tests. Specifically, the best *k* was selected to be maximal index for which the optimal objective function increased more than 5% with respect to previous index and the permutation *p*value did not increase, with this property holding for all smaller indices (*k*^′^<*k*). The permutation test is computed by permuting the phenotype (the mutation counts for each signature in this case) and comparing the objective function value to the ones obtained with the permuted phenotypes. We define the identified module to be significant if the FDR-adjusted *p* value is less than 0.1.

For the analyses with BRCA subtypes, we utilized AIMS subtypes provided in Supplementary Table 18 of Nik-Zainal et al. [25]. The association analyses with gene alteration information were performed with 78, 111, and 64 samples categorized as luminal A, B, and basal subtypes, respectively (there are only 10 samples in HER2 subtype; hence, the results are not reported).

## Results

### Expression analysis to identify biological processes associated with mutational signatures

In order to identify biological processes associated with individual signatures, we clustered gene expression-signature correlation profiles as described in the “Methods” section. To obtain a bird’s eye view, we first used all genes whose expression is correlated with at least one signature (Fig. 2a and Additional File 1: Fig. S1; see the “Methods” section). Next, to obtain a finer scale expression modules related to DNA repair, we zoomed in on genes involved in Gene Ontology DNA metabolic process (Fig. 2b).

The first striking observation is the similarity of gene expression patterns among both variants of Signatures 3 and 13 and all other cloud signatures (2C and 8C). Since Signatures 3 and 13 are considered to be associated with homologous recombination deficiency and APOBEC activity respectively, in what follows we refer to this group of signatures as HRD-APOBEC signature group. Note that Signature 2 is also known as an APOBEC-related signature but the group includes only Signature 2C but not 2D. Below, we will discuss insights obtained for the age-related signatures and the APOBEC signatures and also provide independent supporting evidence from literature. Given expression correlation similarity within the members of the HRD-APOBEC group (all positively correlated with cell cycle, DNA repair, and immune response), we defer the analysis of this group to the next section where we look at this group through the lenses of mutated subnetworks.

#### The expression correlation analysis reveals important differences between the APOBEC signatures

Surprisingly, among 4 APOBEC-related signatures (Signatures 2C/D and 13C/D), Signature 2D has strikingly different correlation patterns compared to the remaining three APOBEC signatures. APOBEC activities are considered to be related to immune response. While the expression correlation patterns of all other APOBEC signatures are consistent with such understanding, Signature 2D exposure level has slightly negative correlation with immune response (Fig. 2a, aC6). This is consistent with our previous observation that there is no positive correlation between Signature 2D and APOBEC expression [9].

In addition, Signature 2 exposure level either is not correlated (2D) or has a weak correlation (2C) with the cluster enriched with translesion synthesis (Fig. 2, aC7 and mC4) whereas both Signatures 13C and 13D show positive correlation. This last observation supports the previous claim that the difference between Signatures 2 and 13 is related to differences in the repair mechanism [36]. Specifically, it has been suggested that mutations in Signature 13 emerge when lesions created by APOBEC activity are repaired by DNA translesion polymerase, which inserts “C” opposite to the damaged base while Signature 2 occurs when the damaged base is simply paired with “A”.

#### Clock-like signatures 1D and 5D have different expression associations suggesting differences in their etiology

Although weaker than the correlation with the HRD-APOBEC Signature group, two clusters enriched in cell cycle function are positively correlated with Signature 1D (Fig. 2a, aC4 and aC5), which is consistent with the previous observation that Signature 1 is associated with aging [26] and thus postulated to be correlated with the number of cell divisions. Consistent with this interpretation, many cancer types with high level of Signature 1 are derived from normal epithelia with high turnover such as the stomach and colorectum [26].

On the other hand, Signature 5D is not positively correlated with the expression of cell cycle genes despite the fact that Signature 5 is also considered to be a clock-like signature. This suggests that accumulation of mutations attributed to Signature 5 is related to the exposure to naturally occurring environmental/external processes. Interestingly, Signature 5D has a positive correlation with the cluster enriched in oxidative processes (Fig. 2a, aC1) and the cluster enriched in nucleotide excision repair (NER) pathway (Fig. 2b, mC1). The accumulation of oxidation base lesions is also assumed to be age-related [47], suggesting that Signature 5 might be related to oxidative damage. NER pathway is involved in neutralizing oxidative DNA damage [48], and Signature 5 has been also associated with smoking [49], which itself is associated with oxidative damage. Indeed, Signature 5 was linked to the NER pathway in a recent study [50]. Finally, comparative analysis of Signature 5 mutation rates in various types of kidney cancers supports the hypothesis that continuous exposure to ubiquitous metabolic mutagens may underlie Signature 5 mutations [26].

The positive correlation of Signature 1 with the expression of cell cycle genes and lack of such correlation for Signature 5 may explain the stronger association of Signature 5 with the age of patients than Signature 1 in breast cancer [9, 26] because cancer-related cell division might obscure the association of Signature 1 with a patient’s age.

### Identifying mutated subnetworks associated with mutational signatures

The analysis of expression correlation clusters revealed different biological processes associated with some signatures, but the signatures in the HR-APOBEC group have largely similar expression patterns and require further investigation. Complementary to the expression analysis, we next searched for possible associations with subnetworks of mutated genes. Some mutational signatures can arise due to endogenous causes; aberrations in genes responsible for different DNA repair mechanisms can lead to the malfunctioning of the corresponding repair process, leaving errors not repaired and in turn generating specific patterns of mutations. We applied NETPHIX, a method to identify phenotype-associated subnetworks, which can help to uncover a subnetwork of genes whose alterations are potentially causative of specific mutational signatures directly or indirectly. Note that not all mutational signatures have such association with mutated pathways. Mutational signatures arising from environmental exposure, age, or other external factors are not necessarily expected to have casual associations with mutated subnetworks.

**Fig. 3:**
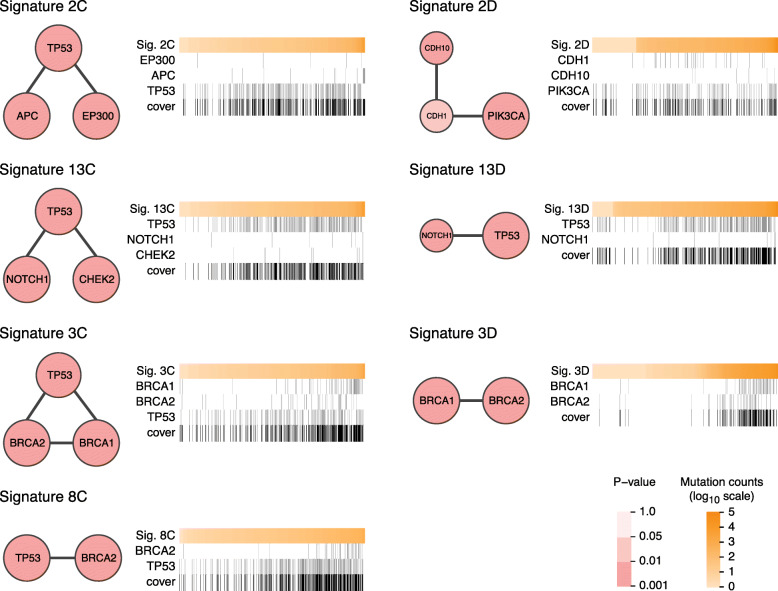
Subnetworks identified by NETPHIX. Panel for each signature consists of a network view of a module (left) and a heatmap showing an association of module gene alterations with signature strength across patients (right). The network node size indicates the gene robustness (regarding NETPHIX results for different random initialization runs of SIGMa), while the darkness of red color represents its individual association score (empirical *p*value based on phenotype permutation test). Each heatmap shows the number of mutations attributed to a given signature for all patients (orange; top row; log_10_ scale) sorted from low to high (columns). For each gene in the module, gene alteration information observed in each patient is shown in gray, while patients not altered are in white. The last row shows the alteration profile of the entire subnetwork in black. Only subnetworks significant in phenotype associations for mutational Signatures 2C, 2D, 13C, 13D, 3C, 3D, and 8C are shown; results for Signatures 1D and 5D were not significant

Figure 3 shows all statistically significant subnetworks (phenotype permutation test; see the “Methods” section) identified by NETPHIX and their alteration profiles. See the “Methods” section (“Mutation analysis” section) for how the module for each signature was selected. The extended subnetworks obtained with less stringent cutoffs are shown in Additional file 1: Fig. S2.

As expected, no modules are found to be significantly associated with the age-related signatures 1D and 5D. This is consistent with the current understanding that these signatures can accumulate due to naturally occurring processes. In addition, consistent with the previous studies that linked the genes underlying the HRD to Signature 3 in breast cancer [51], the subnetworks identified for Signature 3 C/D contain BRCA1 and BRCA2 genes, two important genes in HR-mediated double-strand break (DSB) repair.

The agreement of the modules identified by NETPHIX with the current knowledge confirms its ability to correctly infer mutated subnetworks associated with signatures.

Encouraged by the results, we examined the remaining subnetworks identified by NETPHIX. Among statistically significant modules, TP53 was included in all modules associated with cloud signatures. TP53 is known to play a crucial role in DNA damage responses, including DSB repair. We note that its dysfunction could contribute to increased mutation burden and in turn to the emergence of cloud mutations independently of mutagenic processes underlying individual signatures. However, whether or not TP53 mutations are causal or are a result of yet another mutagenic process cannot be concluded from this study. Complicating this picture, a recent study demonstrated that p53 controls the expression of the DNA deaminase APOBEC3B suggesting a possible mechanism by which mutations in p53 can promote APOBEC expression [52] and thus APOBEC-related mutations. Hence, the reason for the strong association of TP53 with cloud mutational signatures requires further investigation.

Compared to the modules obtained from expression analysis, the analysis with genetic alterations offers a better differentiation among the signatures in the HRD-APOBEC group. While most of the signatures in the group contain TP53, they also include different genes in the modules. In the subnetworks associated with Signatures 13 C/D, TP53 is accompanied by NOTCH1; NOTCH pathway regulates many aspects of metazoan development, including the control of proliferation and differentiation. CHEK2 is selected in addition to TP53 and NOTCH1 for Signature 13C. CHEK2 is a tumor suppressor regulating a cell cycle checkpoint and mutations in the gene confer an increased risk for breast cancer [53, 54]. CHEK2 plays multiple roles in DNA damage response [55], including DSB repair in the emergence of clustered APOBEC-related mutations.

In the subnetwork associated with Signature 2C, TP53 is accompanied by APC (Adenomatous Polyposis Coli), which is a tumor-suppressor gene frequently mutated in colorectal cancer (CRC) and involved in the Wnt signalling pathway. A recent study linked APC to several DNA repair mechanisms, including the base excision repair (BER) pathway [56], DSB repair [57], and genomic stability [58, 59].

Finally, the subnetwork for Signature 2D (dispersed, APOBEC-related signature) consists of PIK3CA, CDH1, and CDH10 genes and is completely different from the subnetworks corresponding to the cloud variant of Signature 2 and other HR-APOBEC-related signatures. Previous studies have found that some recurring mutations in PIK3CA are consistent with Signature 2 and may result from APOBEC activities [14, 60]. However, our analysis associated PIK3CA mutations with Signature 2 even after removing point mutations attributed to Signature 2, suggesting a more complex relation between Signature 2 and PIK3CA mutations.

In addition to PIK3CA, the subnetwork associated with Signature 2D has two Cadherin genes: CDH1 and CDH10. Cadherins are important in the maintenance of cell adhesion and polarity, and alterations of these functions can contribute to tumorigenesis. CDH1 germline mutations have been associated with hereditary lobular breast cancer [61] and hereditary diffuse gastric cancer [62, 63], while a recent study linked mutations in CDH1 and PIK3CA to the immune-related invasive lobular carcinoma of the breast [64]. In breast cancer, mutations in CDH1-PIK3CA module are mutually exclusive with mutations in TP53 and are strongly enriched in Luminal A subtype [65]. Indeed, our analyses of individual subtypes show that the association of a PIK3CA module with Signature 2D is significant only with Luminal A subtype (Additional file 1: Table S1). Interestingly, the module identified in Luminal A contains, in addition to PIK3CA, PTEN gene which is known to be a negative regulator of the PIK3CA [66]. This, combined with the differences in expression correlations noted in the previous section, suggests that the etiology of Signature 2D is different from the other APOBEC mutational signatures (Signatures 2C and 13)

## Discussion

In order to gain insights into the etiology of mutational processes in cancer, we propose two complementary computational approaches and apply them to gain insights into the etiology of mutational processes in breast cancer. Both approaches leverage the idea of network-level association of mutation signatures with gene networks and pathways but differ in the type of utilized data and mathematical formulation. The first approach uses gene expression data; the second approach is focused on the identification of subnetworks of genes whose alterations are associated with each signature.

The expression correlation-based approach allowed us to uncover important differences between clock-like signatures. Clock-like signatures can occur from life-long exposure to naturally occurring mutagenic processes, thus related to aging. The most prominent clock-like signatures are Signatures 1 and 5. Signature 1, a relatively well characterized clock-like signature, is considered to be the result of an endogenous mutational process related to spontaneous deamination of 5-methylcytosine. Each cell division provides an opportunity for such mutations to occur. This explains why many cancer types with high mutation rates of Signature 1 are derived from normal epithelia with high turnover [26]. The correlation of Signature 1 mutation counts with the expression level of cell cycle genes observed in this study provides further supports for this explanation. The etiology of Signature 5 was less clear. Our expression-based analysis revealed that, differently from Signature 1, Signature 5 is not positively correlated with the expression of cell cycle genes. Instead, we found an association of Signature 5 with oxidation process. This observation is consistent with several previous findings. In particular, our findings support the hypothesis that cell proliferation rate may not be a major factor for Signature 5 [26]. In addition, accumulation of oxidation base lesions is assumed to be related to aging [47] as well as smoking, while the association of Signature 5 with smoking was observed in a previous study [49]. More supporting evidence is provided by the association of Signature 5 with the nucleotide excision repair (NER) pathway which was shown to be involved in neutralizing oxidative DNA damage [48]. These results support the view that the correlation of Signature 5 with age is related to a continuous exposure to an environmental/metabolic mutagen.

While expression-based analysis was very valuable for understanding the differences between Signatures 1 and 5, many signatures especially in the HRD-APOBEC signature group exhibit similar expression correlation patterns. The mutated pathway analysis provided additional insights into the differences among these signatures. In particular, both cloud and dispersed Signature 3 are associated with BRCA 1/2 genes while the subnetwork associated with Signature 3C additionally contains TP53. The results of mutated subnetwork analysis also revealed the association of mutations in tumor-suppressor APC for two different cloud signatures (Signature 2C and Signature 8C with a lenient cutoff) and NOTCH1 mutations for both variants of Signature 13.

In order to increase the probability that inferred mutated subnetworks are causal, we removed the mutations attributed to the signature of interest. This eliminates the possibility that the mutations resulted directly from the mutagenic process underlying the signature although it still does not guarantee causality. In particular, the consistent presence of TP53 in the subnetworks associated with cloud signatures makes it tempting to speculate that mutations in TP53 generally increase the mutation rates leading to an increase in cloud mutations. However, other indirect reasons for this association cannot be ruled out. Our analysis also showed unique properties of Signature 2D relative to the remaining APOBEC signatures. This signature is the only signature associated with PIK3CA and not TP53. Previous studies have found that several recurring mutations in PIK3CA are consistent with Signature 2 [14, 60]. However, our analysis indicates that even after removing mutations attributed to Signature 2, the association between PIK3CA mutations and Signature 2D remains. Another known cancer gene present in this subnetwork is CDH1. CDH1 was previously linked to hereditary lobular breast cancer [67] and hereditary diffuse gastric cancer and in particular, about 40% of hereditary diffuse gastric cancer patients are found to have mutations in CDH1 [62, 63]. Invasive lobular carcinoma is characterized by a unique immune signature [68] which might provide additional insights to the etiology of Signature 2. Our previous studies with breast cancer demonstrated that mutations in CDH1-PIK3CA module are mutually exclusive with mutations in TP53 and are enriched in Luminal A subtype [65]. Consistent with the observation, the subtype-specific analysis using NETPHIX indicated that the association between signature 2D and subnetwork involving PIK3CA is particularly significant in the Luminal A subtype. Importantly, the module identified with samples in Luminal A subtype contains PTEN (in addition to PIK3CA), a known negative regulator of PIK3CA [66]. These results suggest that the relation between Signature 2 mutations and the activation of PI3K pathway might be more complex than previously suggested.

Although our goal in this study was to investigate the genomic causes of mutational signatures regardless of cancer subtypes, we also performed the analysis for each subtype separately to examine the potential differences between subtypes. Table S1 (Additional file 1) shows the subnetworks associated with each subtype. While generally consistent with the results using all samples, the results based on individual subtypes suggest that some associations are subtype specific and, as exemplified by the discussion of the PI3K-PTEN pathway above, can provide additional insights to the relation between mutagenic processes and mutated pathways.

## Conclusions

Patterns of somatic mutations in a cancer genome can shed light on mutagenic processes acting on the genome. However, uncovering specific mutagenic processes underlying a given pattern of mutations is challenging. Previous studies demonstrated that network-centric approaches can be helpful for finding genotypic causes of diseases, classifying disease subtypes, and identifying drug targets [19]. In addition, a recent study demonstrated that, within the same cancer type, different gene modules can be enriched in diffident mutational signatures [23]. However, a broader utility of network-based approaches for understating of mutagenic processes in caner was yet to be demonstrated. To fill this gap, we developed two complementing computational approaches and performed the first network-level association analysis of mutation signatures with dysregulated pathways. Based on gene expression data, we identified gene modules whose expression correlates with mutation counts attributed to mutational signatures. Further analysis of these modules provided important insights into the mutagenic processes underlying specific signatures. Complementing expression analysis, we developed an ILP-based method to identify subnetworks of genes whose alterations are associated with each signature. This analysis provided information about potential differences in the etiology of the signatures that could not be gained from the expression analysis alone.

Taken together, our study demonstrates the utility of these two complementary approaches for studying mutational signatures in cancer and provided several new insights into the etiology of mutational signatures.

## Supplementary information

**Additional file 1** Supplemental methods, supplemental figures S1 and S2, and supplemental table S1.

**Additional file 2** Supplemental table S2: genes and GO terms for expression correlation modules.

**Additional file 3** Supplemental table S3: genes and GO terms for expression correlation modules, dNA metabolic genes only.

## Data Availability

The somatic mutations in the cohort of 560 breast cancer (BRCA) whole genomes (single base substitutions and small indels) were downloaded from the ICGC data portal (release 22) [39]. For the expression-based association with signatures, we used the normalized gene expression data for 266 BRCA patients from Supplementary Table 7 of Nik-Zainal et al. [25]. The biallelic inactivation data was collected from Supplementary Tables 4a and 4b of Davies et al. [2]. For functional interactions among genes, we used the data downloaded from STRING database version 10.0 [46]. The mutation counts for signatures (using SigMa) and gene-level mutation tables are available at https://github.com/yooah/NetSig. The source code and the datasets used for and generated during this study are available at the Github site [40].
