# Supplementary material for: Network-based approaches elucidate differences within APOBEC and clock-like signatures in breast cancer
Source: Genome Med. 2020 May 29;12:52. doi: 10.1186/s13073-020-00745-2 (PMC7260830; doi:10.1186/s13073-020-00745-2)
Supplement: Supplementary file 1 — Additional file 1 Supplemental methods, supplemental figures S1 and S2, and supplemental table S1. [file 13073_2020_745_MOESM1_ESM.pdf]

Supplementary Materials

Supplemental Methods

NETPHIX Method

Our analysis for the association of gene alteration information utilized NETPHIX, which was developed to identify network based associations for continuous cancer phenotypes. While the algorithm can deal with more general cases, we used a simple version of NETPHIX, which identifies subnetworks associated with an increased level of phenotypes. See (Kim et al. 2019) for the full details of NETPHIX algorithm.

The optimization problem is formally defined as follows. We are given a graph $G = (V, E)$, with vertices $V = \{1, \ldots, n\}$ representing genes and edges E representing interactions among genes. Let P denote the set of m patients (or cell lines). For each sample $j \in P$, we are also given a phenotype profile value $w_j \in \mathbb{R}$ which quantitatively measures a phenotype (e.g., mutation counts in our study). Let $P_i \subseteq P$ be the set of patients in which gene $i \in V$ is altered. We say that a patient $j \in P$ is *covered* by gene $i \in V$ if $j \in P_i$ i.e. if gene i is altered in sample j. We say that a sample $j \in P$ is *covered* by a subset of genes (or vertices) $S \subseteq V$, if there exists at least one vertex v in S such that $j \in P_v$.

The goal is to identify a connected subgraph S of G of at most k vertices such that the sum of the weights of the samples covered by S is maximized. The weights are computed based on mutation counts. Since we are interested in functionally complementary mutations, we also penalize coverage overlap when a sample is covered more than once by S by assigning a penalty p_j for each of the additional times sample j is covered by S. Let $c_S(j)$ be the number of times element $j \in P$ is covered by S. For a set S of genes, we define its weight $W(S)$ as:

$$W(S) = \sum_{j \in \cup_{s \in S} P_s} w_j - \sum_{j \in \cup_{s \in S} P_s} (c_S(j) - 1) p_j \qquad (1)$$

Thus, we define the optimization problem as follows: Given a graph G defined on a set of n vertices V, a set P, a family of subsets $P = \{P_1, \ldots, P_n\}$ where for each i, $P_i \subseteq P$ is associated with $i \in V$, weights w_j and penalties $p_j \geq 0$ for each sample $j \in P$, find the subset $S \subseteq V$ of $\leq k$ connected vertices maximizing $W(S)$.

We then formulated the problem as integer linear programming (ILP) as follows and solved it to optimality with CPLEX.

$$z(q) = \max \quad \sum_j (w_j + p_j) z_j - \sum_j p_j y_j \tag{2}$$

$$\text{s.t.} \sum_i x_i \leq k, \tag{3}$$

$$y_j = \sum_{i:j \in P_i} x_i, \qquad \forall j \tag{4}$$

$$y_j \geq z_j, \qquad \forall j \tag{5}$$

$$z_j \geq y_j / k, \qquad \forall j \tag{6}$$

$$x_i, z_j \in \mathbb{B}, y_j \in \mathbb{D} \qquad \forall i, j \tag{7}$$

$$\sum_{l:il \in E} x_l \geq C(k-1)(x_i - 1) + C\left(\sum_{l \in V} x_l - 1\right) \qquad \forall i \in V \tag{8}$$

Let x_i be a binary variable (denoted with $x_i \in \mathbb{B}$) equal to 1 if gene $i \in V$ is selected and $x_i = 0$ otherwise. Let z_j be a binary variable equal to 1 if sample j is covered by a gene i and 0 otherwise. Let y_j denote the number of genes in I that cover sample j in the solution. Finally, let w_j be the weight of sample j and p_j be the penalty for sample j. Although the general problem is NP-hard, we obtained the optimal solution to the ILP instances using CPLEX. We ran the program with $k = 1...7$ and the statistical significance of the selected modules was then assessed with permutation tests.

Construction of Gene Alteration Table

The gene level alteration information for the input to NETPHIX is constructed by utilizing all somatic point mutations and small indels for the same 560 patients data. In general, we defined a gene g to be altered for a patient p if it has at least one "valid" mutation in the genomic region of g for p. The definition of "valid" mutations can be different for each signature as we further refined the information by removing mutations attributed to the signature. For example, the input alteration table used for the association with Signature 2 is constructed after removing all somatic mutations assigned to Signature 2. Formally, for the alteration table ALT_i used for association with Signature i, a gene g in ALT_i is defined to be altered only if it has at least one non-silent mutation in the genomic region of g that is

not attributed to Signature i. For ALT_3 and ALT_8, we additionally removed all indels as these signatures are believed to lead to a high burden of indels. Finally, we augmented the alteration table if the gene is annotated as being biallelic inactivated (Supplementary Table 4a and 4b from (Davies et al. 2017)).

References

Kim, Y.-A. et al. (2019). "Identifying Drug Sensitivity Subnetworks with NETPHIX". In: *bioRxiv*. DOI: 10.1101/543876. eprint: https://www.biorxiv.org/content/early/2019/02/08/543876.full.pdf.

Davies, H. et al. (2017). "HRDetect is a predictor of BRCA1 and BRCA2 deficiency based on mutational signatures". In: *Nature Medicine* 23.4, pp. 517–525. DOI: 10.1038/nm.4292.

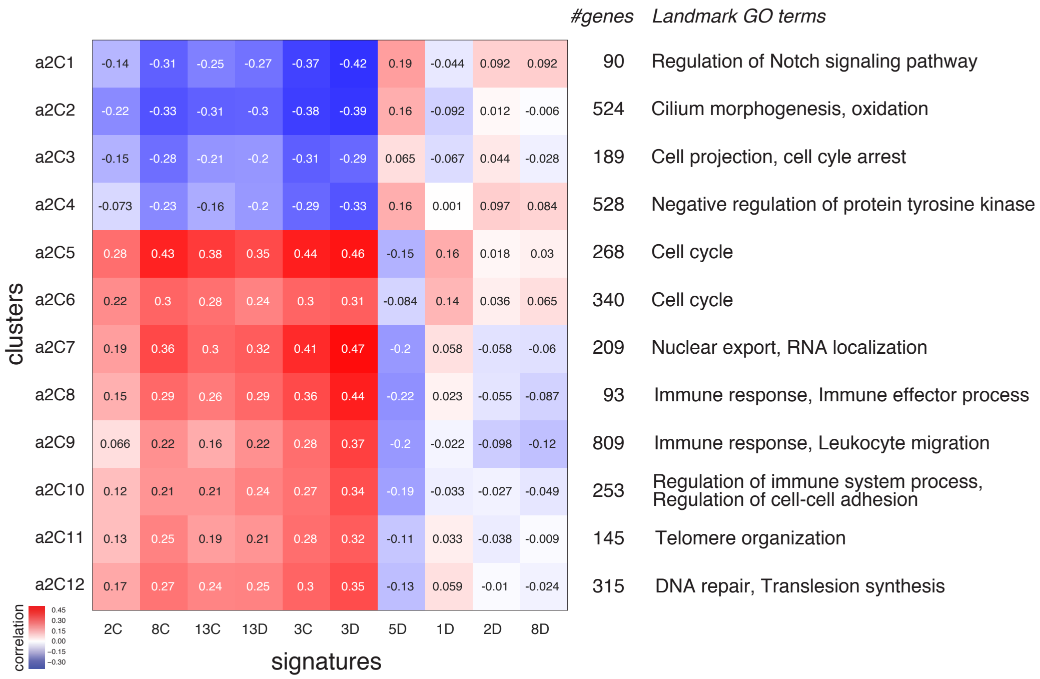

Figure S1: **Gene expression correlation modules (refers to Fig. 2).** Clustering of all genes significantly correlated with at least one of the signatures. This shows a more fine-grained clustering (12 clusters) than in Fig. 2. A heatmap of mean expression correlation for each cluster and signature (left), number of genes in each cluster (middle), and representative GO terms enriched in each cluster of genes (right) are shown.

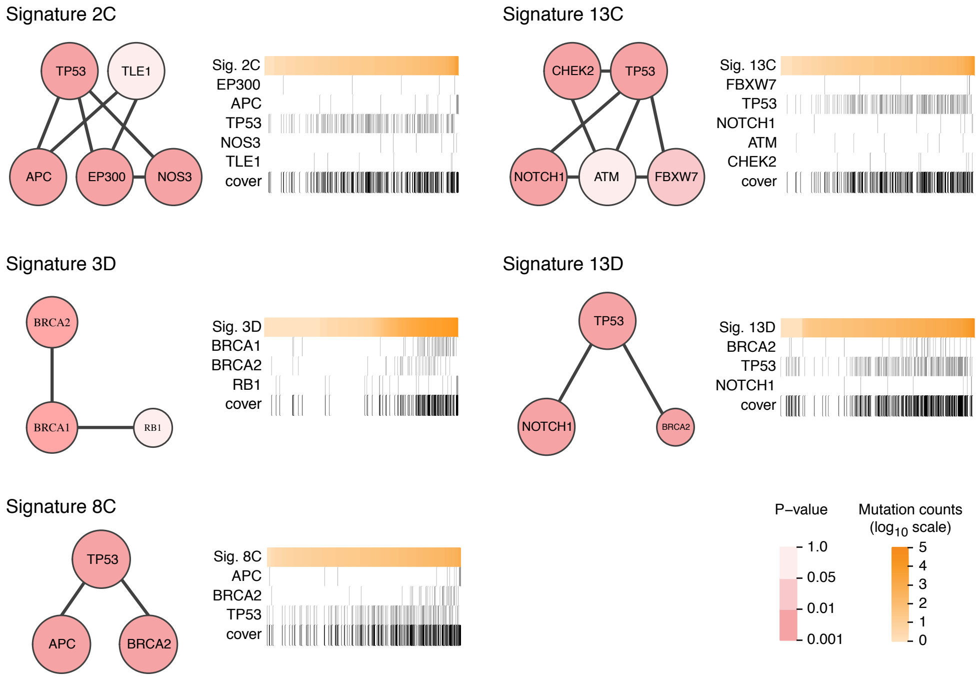

Figure S2: **Subnetworks identified by NETPHIX using less stringent cut-off (refers to Fig. 3).** The best m (the module size) using less stringent cut-offs was selected as maximal index for which the optimal objective function increased more than 1% with respect to previous index and the phenotype p-value did not increase. Panel for each signature consists of a network view of a module (left) and a heatmap showing the association of selected gene alterations with signature strength across patients (right). The network node size indicates the gene robustness (regarding NETPHIX results for different random initialization runs of SigMa) while the darkness of red color represents its individual association score (p-value). Each heatmap shows the number of mutations attributed to a given signature for all samples (orange; top row; $\log_{10}$ scale) sorted from low to high (columns). For each gene in the module, gene mutations observed in each sample caused by other signatures are shown in gray, while samples not altered are in white. The last row shows the mutation profile of the entire subnetwork in black. Only subnetworks that changed with respect to the normal cut-offs (see Fig. 3 and Materials and Methods) are shown. Results for Signatures 2D and 3C did not change with respect to the normal cut-offs and results for Signatures 1D and 5D stayed insignificant (the FDR adjusted p-value above 0.1).

Table S1: **Subnetwork associated with mutational signatures for each subtype**

**Signature**	**Subtype**	**Subnetwork**	***P*-value**
2C	Lum B	APC, TP53, SMAD4, PTEN	0.004
2D	Lum A	PIK3CA, PTEN	0.0049
3C	Lum B	BRCA2, TP53, MAP9	0.001
3C	Basal	BRCA1, BRCA2	0.023
3D	LumA	BRCA1, ARID1A, BRCA2, TP53, NF1	0.002
3D	LumB	BRCA2, TP53, MAP9	0.001
3D	Basal	BRCA1, BARD1, BRCA2, FANCA	0.002
8C	LumB	BRCA2, TP53, KRT19	0.002
13C	LumA	CASP8, TP53, AR, SIN3A, HDAC2	0.023
13C	LumB	CREBBP, BRCA2, TP53	0.003
13D	LumA	HIF1A, BRCA2, TP53, ATM, HDAC2	0.021
13D	LumB	CREBBP, BRCA2, TP53	0.001
